# Ozone Decreased Enteric Methane Production by 20% in an *in vitro* Rumen Fermentation System

**DOI:** 10.3389/fmicb.2020.571537

**Published:** 2020-11-02

**Authors:** Lucy Zhao, Eleonora Caro, Devin B. Holman, Katherine E. Gzyl, Peter J. Moate, Alex V. Chaves

**Affiliations:** ^1^Sydney School of Veterinary Science, Faculty of Science, The University of Sydney, Camperdown, NSW, Australia; ^2^Department of Agricultural, Forest and Food Sciences, University of Turin, Turin, Italy; ^3^Lacombe Research and Development Centre, Agriculture and Agri-Food Canada, Lacombe, AB, Canada; ^4^Agriculture Victoria Research, Ellinbank, VIC, Australia; ^5^Centre for Agricultural Innovation, School of Agriculture and Food, Faculty of Veterinary and Agricultural Sciences, The University of Melbourne, Melbourne, VIC, Australia; ^6^School of Life and Environmental Sciences, Faculty of Science, The University of Sydney, Camperdown, NSW, Australia

**Keywords:** enteric, methanogenesis, rumen, cattle, O_3_, CH_4_ (methane)

## Abstract

Ozone (O_3_) is volatile, highly oxidative, and has theoretical potential to reduce ruminant enteric methanogenesis by interactions between archaea and bacteria, and substrate and oxygen. The effects of O_3_ on the rumen microbiota, fermentation parameters, and CH_4_ emissions were studied through *in vitro* fermentation using a RUSITEC apparatus with O_3_ dissolved in the salivary buffer. The substrate consisted of maize silage or grain concentrates, and the treatments were (1) control (no O_3_) and (2) O_3_ at 0.07 ± 0.022 mg/L in the buffer. A 4-day adaptation period followed by a 6-day experimental period was used for measuring gas production and composition, as well as fermentation characteristics, which included ruminal volatile fatty acids (VFA) and liquid- and solid-associated microbial communities. Ozone treatment decreased total gas production by 15.4%, most notably CH_4_ production by 20.4%, and CH_4_ gas concentration by 5.8%, without compromising dry matter digestibility (DMD) of either maize silage or grain concentrates. There were no significant effects of O_3_ treatment on VFA production or pH. Ozone treatment reduced the relative abundance of methanogens, particularly *Methanomicrobium*. This study demonstrates the potential use of O_3_ as a method to reduce ruminant enteric methanogenesis.

## Introduction

Ruminant CH_4_ production is notoriously one of the largest contributing factors toward greenhouse gas (GHG) emissions, accounting for 50% of emissions from the agricultural sector in Australia, and 7% to total Australian GHG emissions (Hook et al., 2010; Duarte et al., 2017b). Research toward methods to reduce CH_4_ emissions is increasingly valuable with increasing worldwide demand for meat and dairy products; however, minimal progress has actually been achieved (Martin et al., 2010).

Studies have shown that manipulation of feed directly or through additives has been an effective strategy (Beauchemin et al., 2008; Hristov et al., 2013; Teoh et al., 2019) in reducing CH_4_ production. The rumen microbiome produces volatile fatty acids (VFA) from otherwise non-digestible plant polysaccharides and these VFA are the main source of energy to ruminants (Dijkstra, 1994; Henderson et al., 2015). Ruminal archaea, namely, the methanogens, are sensitive to changes in their environment, particularly oxygen availability, and produce CH_4_ as a metabolic by-product under hypoxic conditions (Issazadeh et al., 2013; Patra et al., 2017). Indeed, Moate et al. (2019) have recently shown that in comparison to non-fistulated cows, rumen-fistulated cows have a lower CH_4_ yield, presumably due to inhibition of the methanogenic populations by oxygen.

One common method of disinfecting water is ozonation (also known as O_3_ disinfection), using O_3_ gas to destroy bacteria and viruses (Remondino and Valdenassi, 2018). Ozone gas (O_3_) is formed upon collision of oxygen gas (O_2_) with free oxygen atoms. Currently, O_3_ is widely used in the food industry (Brodowska et al., 2018); however, it has not yet been considered as a dietary additive to mitigate CH_4_ production in ruminants. Methanogens should be inhibited more by O_3_ compared to O_2_, because they are extremely sensitive to oxygen and O_3_ is a more powerful oxidant than O_2_ (Wang et al., 2004). The majority of the rumen microbiome is composed of bacteria and protozoa by mass, with archaea accounting for less than 4% of the microbial community (Matthews et al., 2019). When compounds such as hemiacetal of chloral and starch and halogenated CH_4_ analogs such bromochloro-methane were included in the diets of ruminants, rumen methanogens and CH_4_ production were observed to be severely reduced without negative effects on dry matter intake, dry matter digestibility (DMD) or liveweight gain (Trei et al., 1972; Tomkins et al., 2009; Martinez-Fernandez et al., 2018).

Thus, we hypothesized that (1) if O_3_ was included in an *in vitro* ruminal fermentation process, it would decrease CH_4_ production and (2) would have a CH_4_-inhibiting effect without affecting feed digestibility and other ruminal fermentation parameters. As such, the objective of this study was to evaluate the effect of including O_3_ in an *in vitro* ruminal fermentation system on CH_4_ production, DMD, the rumen microbiota, and other fermentation parameters.

## Materials and Methods

This study was performed in accordance with The University of Sydney Animal Ethics Committee (Approved Protocol Number 2015/835). The cow used for ruminal contents was housed at Corstorphine Dairy, The University of Sydney (Camden Farm Dairy, Cobbitty, NSW, Australia, 34° 04′S; 150°81 69′E).

### Experimental Design and Treatments

A feed consisting of maize silage and concentrates was fed through a RUSITEC apparatus mimicking *in vitro* ruminal fermentation, to investigate the effects of O_3_ supplementation on fermentation parameters, the rumen microbiota and methanogen activity ultimately leading to CH_4_ production. The experiment consisted of a completely randomized design with two treatments: a control and an O_3_-buffered treatment, and three replicates of each over the course of each 10-day experiment (Klevenhusen et al., 2014; Ertl et al., 2015; Sarnataro et al., 2019). The O_3_ treatment contained O_3_ at 0.07 mg/L that was pumped into McDougall’s buffer and then synthesized into the feed, whereas the control consisted of maize silage and concentrates only.

The study was comprised of 2 × 10-day incubation runs, whereby fermentation parameters (including pH, oxidation–reduction potential, total gas production, CH_4_ gas production, and effluent volume production) were measured daily for 6 days following a 4-day adaptation period. These are similar procedures used by Soliva and Hess (2007), Klevenhusen et al. (2014), Ertl et al. (2015), Soliva et al. (2015), and Sarnataro et al. (2019) in Rusitec studies. Effluent samples were collected from days 5–10 for analysis. Solid- and liquid-associated microbes were sampled at days 0, 5, and 10 in each incubation run. DMD was measured on days 6–9 using retained nylon bags. The VFA production was calculated using results from daily (days 0–10) sampling of effluent contents and effluent volume production.

### Preparation of Substrate, O_3_, and Rumen Inoculum

The basis for each feed (control and treatment) consisted of maize silage (50% w/w, 5.5 g DM-basis) and concentrate (“grain”; Table 1) (5.5 g DM-basis for each substrate), weighed into individual porous nylon bags (11 cm × 6.5 cm, pore size 50 μm)—the daily total mass being 11 g (DM basis). Substrate components were derived from Corstorphine farm, The University of Sydney. Details of climate, harvest, processing, and substrate feed analysis are detailed by Teoh et al. (2019).

**TABLE 1:** Chemical composition of substrates used.

	**Crude protein**	**Neutral detergent fiber**	**Crude fat**	**Ash**	**Non-fibrous carbohydrates**
	
	**% in the DM**				
Maize silage	8.4	38.3	3.79	3.23	46.3
Concentrate	15.5	20.8	2.70	8.83	52.2

The ruminal fluid donor, a non-lactating Holstein dairy cow, was fed pasture *ad libitum* (including oaten hay at 6 kg DM/d). The donor was ruminally fistulated and some of the rumen inoculum was collected on the first day of each experimental run, 3 h post-morning feeding. Using cheesecloth for separation, the solid and liquid portions of the ruminal contents were separated and stored in preheated thermos containers, to be immediately transported to the laboratory (Teoh et al., 2019).

The O_3_ was produced by a commercial ozonator (model O3 Series BO3, CSN Global Pty Ltd., Buderim, QLD 4556, Australia) and pumped into the buffer (McDougall’s buffer) using a fish tank aerator (Aqua One Precision 750—3.5 W; 19 kPa). The daily average concentration of O_3_ across the experiment was 0.07 mg/L, measured twice daily for each 10-day run with a dissolved O_3_ meter (SKU-I-2019 model; Ozone Solutions, Hull, IA, United States).

### RUSITEC Fermentation

For each run, the RUSITEC apparatus was set up with six separate fermentation vessels (800 mL each) submerged in a water bath at 39°C. Three of these vessels were intended for the control, and three for the O_3_ treatment. On day 1, each vessel contained three smaller, porous nylon bags in a smaller vessel submerged in approximately 780 mL (until the entire vessel was filled) of the collected liquid ruminal fluid. Of the nylon bags, one contained approximately 70 g wet weight of the collected solid ruminal contents used unprocessed to inoculate the system and another two substrate bags as described above. Each fermentation vessel was equipped with an input port for O_3_-buffered infusion, and an outlet port for the effluent. The fermentation vessels were continuously infused with McDougall’s buffer at a dilution rate of 33 mL/h (control), while the three treatment vessels were continuously infused with the same buffer plus O_3_. To mimic rumination, electric motors moved the contents of the fermentation vessels up and down so as to mix solid and liquid particles and their associated microbes (Teoh et al., 2019).

The nylon bag containing ruminal solids was allowed to incubate for 24 h, before being removed and replaced with two bags: one containing maize silage and one containing concentrates. On day 1, each vessel contained three nylon bags, and on each subsequent day there were four at any given time—and each of these substrate bags was allowed to remain in the fermentation vessel for 48 h (Teoh et al., 2019). The last two bags that were placed in the vessel at day 9 were not used in DM digestibility calculations on day 10 as they had only undergone 24 h of incubation.

### Sample Collection and Analysis

#### Total Gas and CH_4_ Production

Air-tight bags (Plastigas, Linde AG, Munchen, Germany) were connected to the effluent output ports of each fermentation vessel. Each day, the total volume of gas produced by each vessel was recorded using a drum-type gas meter (Terry et al., 2018). Each effluent gas bag was manually pressurized to produce a reading on the meter.

Methane (CH_4_) production was determined daily from day 5 until the end of each 10-day run. To measure daily gas production, 34 mL of gas was collected from each gas bag. Some gases from the previous day’s fermentation may potentially be trapped in the tube connecting the fermentation vessel to the gas sampling bag. To prevent these stale gases from contaminating the gas samples, the first 17 mL from each sample was evacuated, and the remaining 17 mL used for analysis. This retained 17 mL gas sample was transferred into a 10 mL evacuated exetainer (Labco Ltd., High Wycombe, United Kingdom). A 3 mL subsample was taken from the exetainer and CH_4_ percentage was determined by gas chromatography (GC; Agilent model 7890A). The GC conditions used were as described by Teoh et al. (2019). Methane production was calculated by multiplying the total gas volume by the percentage of CH_4_, with correction for temperature and pressure (Duarte et al., 2017b). Results were expressed as mg CH_4_/g DM.

#### Dry Matter Digestibility (DMD), pH, Oxidation–Reduction Potential

Dry matter digestibility was calculated using the contents of the nylon bags (maize silage and concentrates) that had been fermenting for 48 h. The collection of these bags took place on days 6–9, when DNA extraction was not required. Each of the bags was washed in a washing machine set to cold-delicate for 30 min, before being dried at 60°C until a constant weight was obtained (approximately 48 h). The residue weight was recorded and used in the calculation of DMD which was expressed as a percentage (%) of the original DM substrate.

The pH of each vessel was measured daily (during nylon bag exchange) using a pH meter (TPS pH-mV-Temp Meter, Model WP-80) calibrated at 39°C. Redox potential was also measured during this time using an oxidation–reduction potential meter calibrated at 39°C (MW 500 ORP meter range of ±999 mV, accuracy of ±5 mV, Milwaukee Instruments, Inc., Rocky Mount, NC, United States; Ramos et al., 2018).

#### Effluent Volume and Volatile Fatty Acids (VFA) Production

Effluent volume production was measured daily using 2 L glass flasks, connected to the effluent outport of each fermentation vessel and submerged in ice to halt fermentation and microbial growth. Daily effluent was measured using a measuring cylinder and expressed as mL/day. From days 5 to 10, a 2 mL sample of effluent contents was collected for determination of concentrations of VFA. This sample was centrifuged (13,500 × *g* for 2 min at 5°C) and a 1.5 mL subsample of the supernatant was transferred into a 2 mL centrifuge tube and acidified with 0.3 mL metaphosphoric acid (0.20; wt/v). The subsample was then frozen at −20°C until it was analyzed for VFA concentrations using GC (Agilent model 7820A). Crotonic acid was used as internal standard. The settings used were FID set up at 250°C, air flow 300 mL per min, makeup flow (N_2_) ran at 30 mL per min with a capillary column (DB-FFAP, 30 m × 0.32 mm ID × 1 μm). Helium was used as a carrier gas with a flow rate of 30 mL per min. The split inlet was heated to 225°C, and set at 9.526 PSI, with He constant flow 1.5 mL per min, and split ratio 50:1. The oven temperature was programmed to 150°C (held at that temperature for 1 min) and then ramped at 5°C per min to 195°C (held 3 min). Total and individual VFA were estimated daily by multiplying VFA concentration by the volume of effluent.

#### Sequencing and Analysis of the Archaeal and Bacterial 16S rRNA Gene

The V4 hypervariable region of archaea and bacteria was sequenced as previously described (Terry et al., 2018; Teoh et al., 2019) using an Illumina MiSeq instrument and the MiSeq Reagent Kit with 500 cycles (Illumina, Inc., San Diego, CA, United States) according to manufacturer’s instructions. DADA2 v. 1.12.1 (Callahan et al., 2016) in R v. 3.6.1 was used to process and quality-filter all sequences. Briefly, the forward and reverse reads were trimmed to 220 and 200 bp, respectively, merged with a minimum overlap of 75 bp, and chimeras removed. Taxonomy was assigned to the remaining sequences, referred to here as operational taxonomic units (OTUs) at 100% similarity, using the RDP naïve Bayesian classifier and the SILVA SSU database release 132 (Quast et al., 2012). The OTUs classified as chloroplasts, mitochondria, or eukaryotic in origin were removed prior to analyses.

The number of OTUs per sample, Shannon diversity, and inverse Simpson’s diversity indices were calculated in R using Phyloseq 1.28.0 (McMurdie and Holmes, 2013). The bacterial structure was assessed using Bray–Curtis dissimilarities which were calculated with vegan 2.5.6 and the effect of O_3_ treatment was determined using PERMANOVA (adonis2 function) in R with 10,000 permutations. The LAM and SAM samples were assessed separately as they are quite different from each other in terms of microbial diversity and structure (Duarte et al., 2017b).

All 16S rRNA gene sequences were submitted to the Sequence Read Archive under BioProject number PRJNA590488.

### Statistical Analysis

Fermentation data were analyzed using the MIXED procedure of SAS (SAS, Inc., 2020; SAS Online Doc 9.1.4). The model included the fixed effects of O_3_, day, and O_3_ × day interaction. Therefore, the individual fermenter was used as the experimental unit for statistical analysis. The minimum values of Akaike’s information criterion were used to select the covariance structure among compound symmetry, heterogeneous compound symmetry, autoregressive, heterogeneous autoregressive, Toeplitz, unstructured, and banded for each parameter. Treatment means were compared using the least squares mean linear hypothesis test (LSMEANS/DIFF). Significance was declared at *P* ≤ 0.05 and a trend was discussed when 0.05 < *P* ≤ 0.10.

Associations between the relative abundance of the 10 most relatively abundant genera, as well as three methanogenic taxa, and fermentation parameters were assessed using Spearman’s rank correlation coefficient (ρ). *P*-values were adjusted for multiple comparisons the Benjamini–Hochberg method. Fermentation parameters were fit to the non-metric multidimensional scaling (NMDS) ordinations of the Bray–Curtis dissimilarities using the envfit function in vegan with 10,000 permutations. Differentially abundant OTUs between the O_3_ and control treatments were determined using DESeq2 v. 1.24.0 (Love et al., 2014) in R. To account for unequal sequencing depth, the 16S rRNA gene samples were randomly subsampled to 23,900 sequences per sample prior to analysis of the diversity measures and Bray–Curtis dissimilarities. DESeq2 uses a negative binomial generalized linear model and the Wald test to identify differentially abundant OTUs. For the DESeq2 analysis, samples were not randomly subsampled and only OTUs present in ≥25% of the samples were included.

## Results

### Effect of O_3_ on Fermentation Parameters

In comparison to the control, O_3_ treatment approximately doubled the measured oxidation–reduction potential (*P* ≤ 0.01) in the buffer solutions in the AM and PM, but there was no difference in the oxidation–reduction potential in the incubation fluid of the control and O_3_ treatments (Table 2). The addition of O_3_ caused a 15.4% decrease in total gas production (*P* ≤ 0.01), and a 20.4% decrease in CH_4_ production (*P* ≤ 0.01), without compromising DMD of either the maize silage (*P* = 0.81) or concentrates (*P* = 0.44), nor total VFA production (*P* = 0.49) (Table 2). The concentration of CH_4_ (%) was decreased by 5.8% in the O_3_-buffered treatments (*P* = 0.01). Additionally, O_3_ supplementation had no effect on the pH (*P* = 0.87). There were no effects of O_3_ treatment on acetate, propionate, branched-chain VFA, valerate, or caproate production (*P* ≥ 0.23); however, butyrate production (mmol/d) was 7.14% greater (*P* = 0.04) with O_3_ supplementation compared to the control treatment.

**TABLE 2:** Effect of ozone (O_3_) on oxidation–reduction potential (Redox), dry matter disappearance (DMD), gas, methane, and volatile fatty acids (VFA) production.

	**Control**	**O_3_**	**SEM**	***P*-value**
pH	6.82	6.82	0.04	0.87
Redox buffer, AM	140.9	288.4	12.64	<0.01
Redox buffer, PM	160.3	305.2	12.75	<0.01
Redox vessel	−317.4	−319	6.83	0.87
Gas production, mL	1790.8	1515.2	50.06	<0.01
CH_4_, %	5.87	5.53	0.07	0.01
CH_4_, mg/d	75	59.7	2.28	<0.01
DMD concentrate, %	75.9	74.9	0.89	0.44
DMD silage, %	60.1	60	0.52	0.81
Total VFA, mmol/d	9.38	9.75	0.36	0.49
Acetate (A), mmol/d	4.51	4.45	0.16	0.81
Propionate (P), mmol/d	5.37	5.19	0.13	0.31
Butyrate, mmol/d	1.68	1.80	0.04	0.04
BCVFA, mmol/d	0.26	0.25	0.01	0.40
Valerate, mmol/d	2.36	2.27	0.08	0.37
Caproate, mmol/d	0.52	0.47	0.03	0.23
Ratio A:P	0.84	0.86	0.039	0.07

*BCVFA, branched-chain VFA (iso-butyrate + iso-valerate); SEM, standard error of the means.*

### Effect of O_3_ on the Rumen Microbiota

**FIGURE 1:**
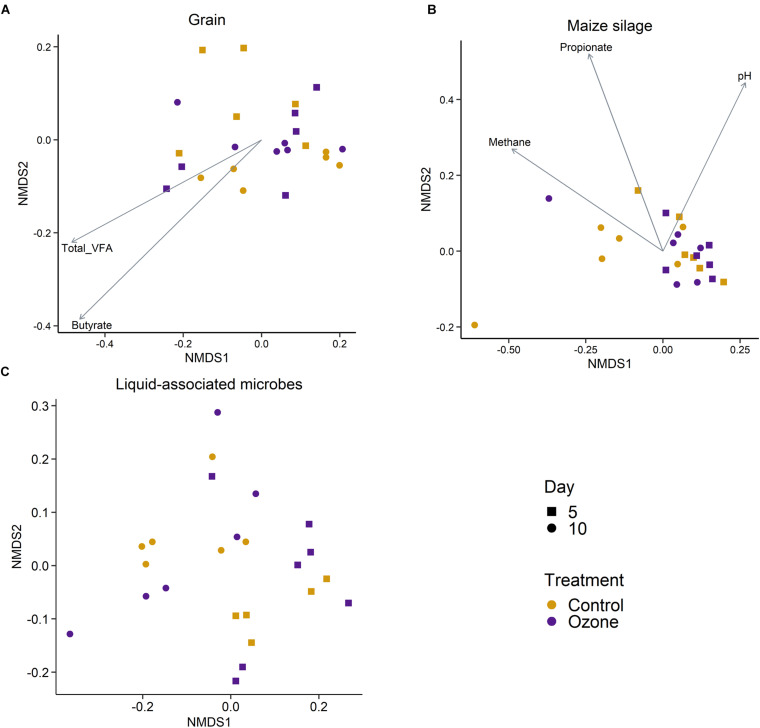
Non-metric multidimensional scaling (NMDS) of the Bray–Curtis dissimilarities for archaeal and bacterial solid-associated rumen samples by ozone treatment and sampling day for the **(A)** grain concentrates and **(B)** maize silage substrates. **(C)** NMDS of the liquid-associated rumen samples by sampling day and ozone treatment. In **(A,B)**, vectors having a statistically significant association (*P* < 0.05) with the ordinations are included. Vector length is proportional to the degree of correlation between the fermentation parameters and the ordination.

A total of 3491 archaeal and bacterial OTUs were identified among all samples with an average sequencing depth of 53,411 ± 1454 SEM sequences per sample. The structure of the rumen solid-associated microbiota (SAM) was strongly affected by substrate (PERMANOVA: *R*^2^ = 0.63; *P* < 0.01; Supplementary Figure S1) and less so by sampling time (*R*^2^ = 0.048; *P* < 0.01; Figures 1A,B). However, the solid-associated rumen samples were not affected by O_3_ treatment within each substrate type (*P* > 0.05). The microbial community structure of the liquid-associated rumen samples (LAM) was also not affected by O_3_ treatment (*P* > 0.05) but there was a time effect (*R*^2^ = 0.25; *P* < 0.01; Figure 1C). Additionally, no differentially abundant OTUs were identified between the control and O_3_ treatments for either the LAM or SAM samples. Among the fermentation parameters, butyrate (*R*^2^ = 0.36; *P* < 0.01) and total VFA (*R*^2^ = 0.28; *P* < 0.05) were significantly associated with the NMDS ordination of the Bray–Curtis dissimilarities for the SAM samples from the grain concentrates substrate. Methane (*R*^2^ = 0.31; *P* < 0.05) and propionate (*R*^2^ = 0.33; *P* < 0.05) concentrations as well as pH (*R*^2^ = 0.27; *P* < 0.05) were significantly correlated with NMDS ordination for the maize silage SAM samples. There were no differences on the alpha diversity indices identified between the O_3_ and control for the LAM (Supplementary Table S1). Further analysis of the SAM microbiota (Table 3) demonstrated that on day 10 the supplementation with O_3_ decreased the number of OTUs (richness) and the Shannon diversity index (*P* = 0.02) compared to the control. On day 5, none of the alpha-diversity metrics were affected by O_3_ treatment (*P* ≥ 0.63).

**TABLE 3:** Effect of ozone (O_3_) on richness (number of OTUs) and diversity indexes of the solid-associated microbe (SAM) samples for each sampling days 5 and 10.

		**Treatment**			**Substrate**			***P*-value**		
**SAM**	**Day**	**Control**	**O_3_**	**SEM**	**Grain**	**Maize silage**	**SEM**	**O_3_**	**Substrate**	**O_3_ × substrate**
Number of OTUs	5	249	234	22.6	171	312	22.0	0.63	<0.01	0.07
Shannon diversity index	5	2.5	2.5	0.13	2.37	2.54	0.133	0.99	0.39	0.10
Inverse Simpsons diversity	5	5.09	5.12	0.539	5.66	4.54	0.514	0.97	0.14	0.14
Number of OTUs	10	302	226	21.1	193	336	21.1	0.02	<0.01	0.80
Shannon diversity index	10	3.15	2.57	0.149	2.51	3.21	0.144	0.02	0.01	0.20
Inverse Simpsons diversity	10	11.68	5.77	3.008	6.30	11.16	2.914	0.20	0.25	0.30

*SEM, standard error of the means.*

Five unique archaeal genera along with unclassified *Methanomethylophilaceae* members belonging to the *Methanobacteria* and *Thermoplasmata* classes were identified in the rumen samples. All of these taxa are methanogens. The large majority of archaeal 16S rRNA gene sequences were identified as unclassified members of the *Methanomethylophilaceae* family (data not shown). The overall relative abundance of these methanogenic taxa was 5.15 ± 0.28% (SEM) and 0.43 ± 0.04% in the LAM and SAM samples, respectively. Ozone treatment decreased the relative abundance of the total methanogen population in the SAM microbiota on day 10 (*P* = 0.02; Table 4), as well as *Methanomicrobium* (*P* = 0.05) and unclassified *Methanomethylophilaceae* (*P* = 0.01). There was no effect of O_3_ treatment (*P* ≥ 0.14) on any of the methanogenic genera in the LAM microbiota (Table 5).

**TABLE 4:** Effects of ozone (O_3_) treatment on the percent relative abundance of methanogenic genera on days 5 and 10 in solid-associated microbes (SAM) samples.

		**Treatment**			**Substrate**			***P*-value**		
**SAM**	**Day**	**Control**	**O_3_**	**SEM**	**Grain**	**Maize silage**	**SEM**	**O_3_**	**Substrate**	**O_3_ × substrate**
*Methanobrevibacter*	5	0.149	0.142	0.0308	0.052	0.239	0.0308	0.88	<0.01	0.72
*Methanosphaera*	5	ND	ND	ND	ND	ND	ND	N/A	N/A	N/A
*Methanomicrobium*	5	0.014	0.018	0.0054	0.008	0.023	0.0041	0.58	<0.01	0.34
*Methanimicrococcus*	5	ND	ND	ND	ND	ND	ND	N/A	N/A	N/A
*Methanomethylophilus*	5	0.002	0.002	0.0015	0.002	0.002	0.0015	0.78	0.78	0.10
UC *Methanomethylophilaceae*	5	0.229	0.220	0.0537	0.136	0.313	0.0537	0.91	0.03	0.37
Total methanogens	5	0.393	0.383	0.0712	0.198	0.578	0.0712	0.92	<0.01	0.38
*Methanobrevibacter*	10	0.077	0.079	0.0192	0.034	0.122	0.0192	0.95	<0.01	0.76
*Methanosphaera*	10	ND	ND	ND	ND	ND	ND	N/A	N/A	N/A
*Methanomicrobium*	10	0.058	0.039	0.0061	0.030	0.068	0.0061	0.05	<0.01	0.14
*Methanimicrococcus*	10	ND	0.0014	0.0009	0.0002	0.0012	0.00086	0.25	0.44	0.44
*Methanomethylophilus*	10	0.002	0.005	0.0015	0.005	0.002	0.0015	0.23	0.18	0.10
UC *Methanomethylophilaceae*	10	0.433	0.268	0.0431	0.222	0.479	0.0431	0.01	<0.01	0.13
Total methanogens	10	0.570	0.392	0.0512	0.290	0.672	0.0512	0.02	<0.01	0.17

*SEM, standard error of the means; ND, not detected; N/A, non-applicable. UC, unclassified.*

**TABLE 5:** Effects of ozone (O_3_) treatment on the percent relative abundance of the methanogenic genera on days 5 and 10 in liquid-associated microbe (LAM) samples.

**LAM**	**Day**	**Control**	**O_3_**	**SEM**	***P*-value**
*Methanobrevibacter*	5	0.319	0.600	0.1323	0.17
*Methanosphaera*	5	0.001	0.009	0.0036	0.15
*Methanomicrobium*	5	0.049	0.042	0.0129	0.72
*Methanimicrococcus*	5	0.004	0.002	0.0016	0.48
*Methanomethylophilus*	5	0.070	0.067	0.0283	0.94
UC *Methanomethylophilaceae*	5	5.71	4.74	0.422	0.14
Total methanogens	5	6.15	5.46	0.363	0.21
*Methanobrevibacter*	10	0.237	0.326	0.0539	0.27
*Methanosphaera*	10	0.001	0.001	0.0010	0.59
*Methanomicrobium*	10	0.291	0.384	0.1452	0.66
*Methanimicrococcus*	10	0.004	0.019	0.0084	0.22
*Methanomethylophilus*	10	0.050	0.051	0.0211	0.97
UC *Methanomethylophilaceae*	10	4.28	3.36	0.544	0.25
Total methanogens	10	4.87	4.14	0.592	0.40

*SEM, standard error of the means; UC, unclassified.*

**FIGURE 2:**
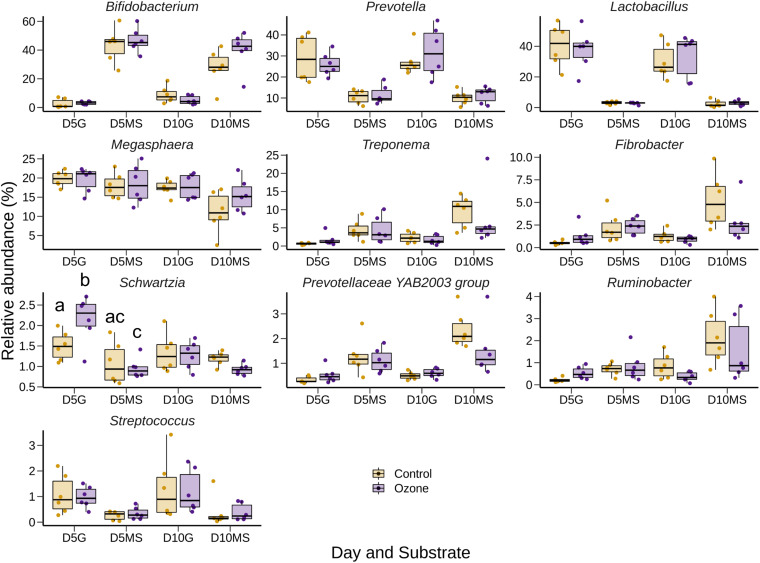
Box and whisker plots of the 10 most relatively abundant bacterial genera in the solid-associated microbe (SAM) rumen samples by ozone treatment and day (D5 or D10) for the grain (G) and maize silage (MS) substrates. The box in the box plots indicates the interquartile range (IQR) (middle 50% of the data), the middle line represents the median value, and the whiskers represent 1.5 times the IQR.

**FIGURE 3:**
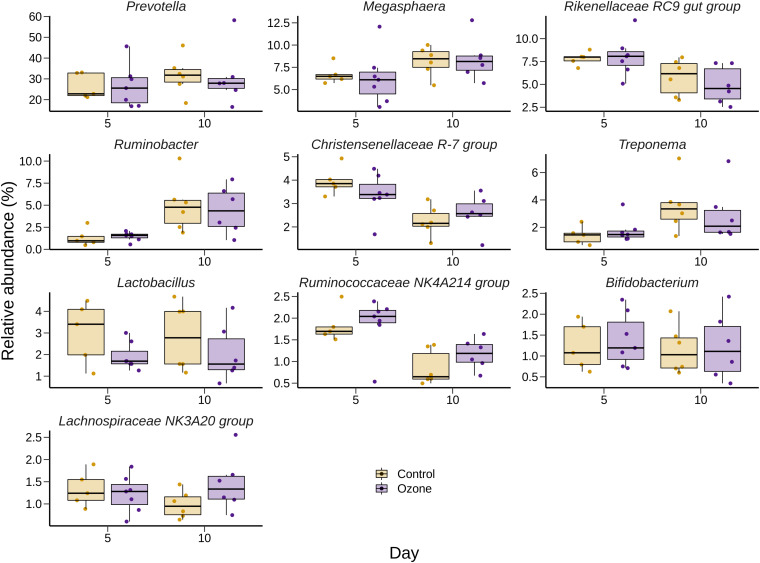
Box and whisker plots of the 10 most relatively abundant bacterial genera in the liquid-associated microbe (LAM) rumen samples ozone treatment. The box in the box plots indicates the interquartile range (IQR) (middle 50% of the data), the middle line represents the median value, and the whiskers represent 1.5 times the IQR.

**FIGURE 4:**
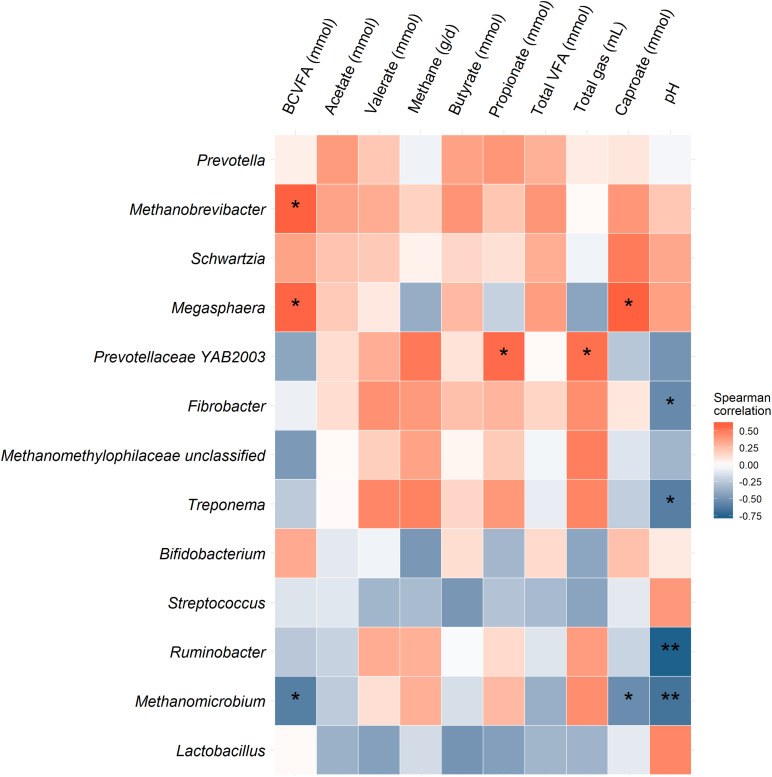
Heatmap of the association between the 10 most relatively abundant bacterial genera and three methanogenic taxa and fermentation parameters based on Spearman’s rank correlation coefficient. Correlations that are statistically significant (*P* < 0.05) are indicated with “**” and those with *P*-values less than 0.10 are denoted with “*”.

The effect of O_3_ treatment on the 10 most relatively abundant bacterial genera on days 5 and 10 in the SAM samples is displayed in Table 6 and Figure 2. Nearly all of these genera differed in relative abundance between the grain concentrates and silage diets; however, only the *Schwartzia* genus was significantly affected by the O_3_ treatment, and only on day 5 in the grain diet. None of the other relatively abundant genera were affected by O_3_ supplementation. There were no significant differences in the relative abundance of the 10 most relatively abundant bacterial genera in the LAM samples by O_3_ treatment or between the grain concentrates and maize silage diets (*P* > 0.05) (Figure 3). *Prevotella* and *Bifidobacterium* were the most relatively abundant genera in the LAM and SAM samples, respectively. Associations between the 10 most relatively abundant genera as well as the three most relatively abundant methanogenic taxa and fermentation parameters were determined using Spearman’s rank correlation coefficient (Figure 4). Only the relative abundance of *Methanomicrobium* and *Ruminobacter* was significantly associated with any of the parameters (*P* < 0.05) and both were negatively correlated with pH. Some of the positive associations of note (Spearman’s rank correlation coefficient ρ > 0.50; *P* < 0.10) were *Prevotellaceae* YAB2003 with CH_4_ and propionate, *Megasphaera* with BCVFA and caproate, and *Methanobrevibacter* with BCVFA.

**TABLE 6:** Effects of ozone (O_3_) treatment on the 10 most relatively abundant bacterial genera on days 5 and 10 in solid-associated microbe (SAM) samples.

		**Treatment**			**Substrate**			***P*-value**		
**SAM**	**Day**	**Control**	**O_3_**	**SEM**	**Grain**	**Maize silage**	**SEM**	**O_3_**	**Substrate**	**O_3_ × substrate**
*Bifidobacterium*	5	23.05	24.99	2.164	2.91	45.13	2.164	0.53	<0.01	0.65
*Fibrobacter*	5	1.38	1.82	0.318	0.91	2.30	0.318	0.34	0.01	0.50
*Lactobacillus*	5	21.84	20.28	2.777	39.19	2.93	2.747	0.70	<0.01	0.76
*Megasphaera*	5	18.89	18.99	1.201	19.70	18.18	1.017	0.95	0.20	0.80
*Prevotella*	5	19.80	18.74	2.316	27.50	11.04	1.938	0.75	<0.01	0.36
*Prevotellaceae YAB2003* group	5	0.80	0.82	0.137	0.42	1.20	0.136	0.94	<0.01	0.35
*Ruminobacter*	5	0.46	0.69	0.118	0.38	0.78	0.116	0.20	0.03	0.60
*Schwartzia*	5	1.29	1.55	0.127	1.83	1.02	0.127	0.15	<0.01	0.05
*Streptococcus*	5	0.67	0.66	0.133	1.03	0.30	0.133	0.95	<0.01	0.64
*Treponema*	5	2.51	3.05	0.699	1.17	4.39	0.699	0.59	<0.01	0.61
*Bifidobacterium*	10	18.36	22.45	2.805	6.99	33.82	2.805	0.32	<0.01	0.06
*Fibrobacter*	10	3.24	1.88	0.597	1.10	4.03	0.553	0.14	<0.01	0.21
*Lactobacillus*	10	16.44	18.42	2.705	32.09	2.77	2.648	0.61	<0.01	0.70
*Megasphaera*	10	14.27	16.63	1.136	17.58	13.32	1.136	0.16	0.02	0.21
*Prevotella*	10	18.79	21.66	2.091	29.63	10.83	2.091	0.34	<0.01	0.62
*Prevotellaceae YAB2003* group	10	1.43	1.07	0.197	0.55	1.95	0.197	0.21	<0.01	0.11
*Ruminobacter*	10	1.49	0.96	0.286	0.60	1.85	0.286	0.22	0.01	0.91
*Schwartzia*	10	1.26	1.10	0.098	1.31	1.06	0.089	0.27	0.05	0.36
*Streptococcus*	10	0.84	0.79	0.305	1.25	0.39	0.236	0.91	<0.01	0.79
*Treponema*	10	5.95	4.52	1.391	1.99	8.48	1.372	0.49	0.01	0.69

*SEM, standard error of the means.*

## Discussion

It is known that manipulation of diet through changes in composition or additives is an effective strategy to reduce enteric methanogenesis in the ruminant (Beauchemin et al., 2008; Hristov et al., 2013; Teoh et al., 2019). It has been suggested that certain bacterial and archaeal species in the rumen are more susceptible than others to unfavorable conditions (Wintsche et al., 2018). In the present study, inclusion of O_3_ in the buffer during *in vitro* fermentation led to a 15.4% decrease in total gas production, and a 20.4% decrease in CH_4_ production, confirming our first hypothesis. As far as we can ascertain, this is the first study to demonstrate *in vitro*, that ruminal methanogenesis can be inhibited by O_3_.

The O_3_ treatment reduced enteric CH_4_ production without compromising DMD. Although treatment with O_3_ did not significantly alter the bacterial and archaeal microbiota in the LAM samples and had no effect on the SAM bacterial populations, it was associated with a reduction in relative abundance of methanogens in the SAM samples and an increase in the production of butyric acid. In particular, significant effects were observed on both the SAM methanogenic microbiota and bacterial diversity and richness on day 10. Feng et al. (2013) demonstrated similar findings with a decreased methanogen diversity in rice-paddy soil exposed to elevated ground-level O_3_. While the most relatively abundant genera differ between the present study and that of Feng et al. (2013), similar patterns of increasing richness (number of OTUs) over time were observed.

The relative abundance of the *Methanomicrobium* genus, unclassified *Methanomethylophilaceae*, and total methanogenic 16S rRNA gene sequences was significantly reduced on day 10 in the O_3_-treated SAM samples (*P* ≤ 0.05). Similarly, Zhang et al. (2016) indicated a decrease in the abundance of methanogens in both O_3_-sensitive and O_3_-tolerant rice cultivars subjected to elevated ground-level O_3_. In the present study, none of the methanogenic genera in either the rumen solid or liquid samples were affected by the O_3_ treatment on day 5. However, by day 10, the relative abundance of methanogens in the solid samples was reduced by approximately 31%, indicating that the effect of O_3_ on methanogens increased with time. *Methanomicrobium* and other hydrogenotrophs including *Methanobrevibacter* utilize recycled hydrogen and carbon dioxide for methanogenesis. Ozone molecules are highly reactive and when present in the rumen will preferentially utilize H_2_ for conversion into H_2_O, reducing hydrogen ion availability for methanogens to synthesize CH_4_ (Zabranska and Pokorna, 2018). Competition for hydrogen thus has the ability to reduce methanogen activity, as seen in Zhao et al. (2018). A similar reduction in methanogenesis by using this hydrogen-competition mechanism has been achieved with other feed supplements such as nitrate (Zhao et al., 2018). However, this hydrogen competition mechanism is unlikely to be the only mechanism whereby ozone inhibits methanogenesis. As referred to in the introduction, ozone has a general biocidal effect against many types of microorganisms, and given the sensitivity of methanogens to oxygen, it seems that ozone may also have a specific biocidal effect against methanogens, or at least a direct inhibitory effect on methanogenesis (Brodowska et al., 2018; Remondino and Valdenassi, 2018).

*Methanomethylophilaceae* is a newly proposed family within the *Methanomassiliicoccales* order (Gaci et al., 2014). The characterized taxa in this order are hydrogen-dependent methylotrophs that produce CH_4_ through the reduction of methanol or methylamine (Lang et al., 2015) and are relatively abundant in the bovine rumen (Henderson et al., 2015; Holman and Gzyl, 2019). Interestingly, we recently reported a decrease in *Methanomethylophilaceae* members in response to hardwood biochar supplementation in a similar RUSITEC experiment (Teoh et al., 2019). We did not find any strong associations between any of the three most relatively abundant methanogenic taxa and CH_4_ production although the PCR primers used likely do not amplify all 16S rRNA genes found in methanogens. *Methanobrevibacter* was positively associated with BCVFA concentration while *Methanomicrobium* was negatively correlated. This may be related to the fact that *Methanobrevibacter* spp. and *Methanomicrobium* spp. have opposite growth requirements for BCVFA (Tanner and Wolfe, 1988; Miller and Lin, 2002). *Schwartzia* was the only bacterial genus among the relatively abundant genera that was significantly affected by O_3_ treatment and only in the grain substrate diet at day 5. The only species in this genus is *Schwartzia succinivorans* which was originally isolated from the bovine rumen and utilizes succinate as a sole energy source producing propionate (van Gylswyk et al., 1997). *Schwartzia* has also been previously reported to be negatively associated with CH_4_ emissions in cattle (Cunha et al., 2017).

Ozone is a highly reactive free radical with strong positive redox potential enabling it to attack glycoproteins and glycolipids in the bacterial cell membrane resulting in rupture and destruction of the cell (Greene et al., 2012; Megahed et al., 2018). In the present research, O_3_ treatment almost doubled the redox potential in the buffer solution added to the “O_3_” incubations. Methanogenic archaea have unique cell walls composed of pseudopeptidoglycan, or pseudomurein, components that make their cell wall similar to those of Gram-positive bacteria and this may contribute to their sensitivity to antimicrobials (Varnava et al., 2017; Doyle et al., 2019) and destructive agents such as O_3_ that target glycosidic bonds. Friedman et al. (2016) suggested that the redox potential effect is caused by a reduction in the number of anti-reactive oxygen species proteins in the genome of the most abundant methanogens, particularly *Methanomicrobium* spp. Additionally, O_3_ is able to cause a complete loss of function by disruption of essential enzymatic activity, as the disturbance of membrane-bound enzymes, proteins, and DNA leads to cell wall lysis (Komanapalli and Lau, 1996) and ultimately a reduction in methanogenesis and total gas production.

Differences in individual and herd CH_4_ emissions can be attributed to intrinsic factors such as the rumen microbiota and variances in particle retention time in the rumen (Martin et al., 2010), as well as extrinsic factors such as manipulation of feed components including silage derivatives, concentrates, and additives. Methanogenesis is largely dependent on the metabolic properties and function of certain members of the rumen bacterial microbiota, which are in turn dependent on substrate type and availability (Evans et al., 2019). Although forage substrate preparation has been shown to have little effect on fermentation parameters (Duarte et al., 2017a), forage quality is known to impact the rumen microbiota, with cattle having a higher feed conversion efficiency producing approximately 20% less CH_4_ (Nkrumah et al., 2006; Hegarty et al., 2007). Feed used in the present study is based on a combination of maize silage and grain concentrates at 50% w/w on a 5.5 g DM basis, consisting of 11.95% CP and 29.56% NDF. Similar studies have seen a correlation between the use of relatively high-quality forage and a reduction in ruminant CH_4_ production (Ramos et al., 2018); though further investigation is required to demonstrate a causal relationship as other experimental conditions are highly variable (Benchaar et al., 2001).

High concentrate diets with a greater presence of readily fermentable substrates (e.g., starch) compared to high forage diets have resulted in a decrease in CH_4_ emissions (Popova et al., 2011). Starch in grain silages favors propionate production rather than acetate as propionate acts as a sink for hydrogen ions and reduces the availability of H_2_ for methanogens (Beauchemin et al., 2008). Thus, an increase in the abundance of propionate-producing bacteria should reduce methanogenesis through the diversion of hydrogen (Myer et al., 2015). Indeed, a decreased acetate: propionate (A:P) ratio has been observed in numerous feed manipulation studies that have resulted in decreased CH_4_ production (Ramos et al., 2018; Williams et al., 2019). It should also be noted that the A:P ratio tended to be greater (*P* = 0.07) in the O_3_ treatment; this despite a high relative abundance of *Prevotella* spp., some of which are propionate producers (McCabe et al., 2015; Emerson and Weimer, 2017). This also corresponds with the positive association observed between the related *Prevotellaceae* YAB2003 group and propionate concentration. Taken together, our findings that ozone had no effect on the A/P ratio, no effects on the relative abundance of ruminal bacteria, but reduced the total number of methanogens and reduced production of methane indicate that ozone treatment specifically inhibits methanogenesis.

While the present study has shown O_3_ treatment can reduce methanogenesis in bovine ruminal fluid, there are many questions to be answered before this could be implemented as a strategy to reduce CH_4_ emissions from the livestock industries. For example, what would be the best and cheapest way to administer O_3_ to ruminants? Ozonation of drinking water offers a potential solution since substantial production benefits have been realized when using clean drinking water in cattle (Schütz, 2012). It is also unknown whether O_3_ administration reduces enteric CH_4_ production *in vivo*. Results from this study indicate the importance and promise of *in vivo* testing, although certain factors including rumen transit time, individual rumen microbiota composition, and fermentation characteristics will differ between individual cattle. Additionally, does O_3_ administration to ruminants have adverse impacts on their health and production? It is hoped that this experiment may stimulate further research designed to answer these questions and that O_3_ may eventually play a role in reducing enteric CH_4_ emissions from livestock industries.

## Conclusion

The inclusion of O_3_ into a fermenter vessel via buffer decreased total gas production, notably CH_4_ production by 20.4%. It is important to note that neither DMD nor total VFA production was affected. Ozone, upon interaction with pre-existing oxygen in the buffer, doubled the buffer redox potential. There was no effect by O_3_ on other fermentation parameters, with the exception of a 7% increase in butyrate production. Ozone treatment decreased both the richness (number of OTUs) and diversity (Shannon diversity) in the solid rumen samples on day 10 (*P* = 0.02). Also, on day 10, O_3_ treatment decreased the relative abundance of the total methanogen population in the SAM samples, particularly *Methanomicrobium* spp. The decrease in CH_4_ gas production may be explained by disruption of the members of the methanogenic genera; however, further research is required *in vivo* to ensure the safety and efficiency of O_3_ for commercial agriculture.

## Data Availability Statement

The datasets presented in this study can be found in online repositories. The names of the repository/repositories and accession number(s) can be found in the article/Supplementary Material.

## Ethics Statement

The animal study was reviewed and approved by The University of Sydney Animal Ethics Committee (Approved Protocol Number 2015/835).

## Author Contributions

AC designed the studies. LZ, EC, DH, KG, PM, and AC acquired data, read and critically revised drafts for intellectual contents, and approved the final manuscript. LZ, EC, DH, PM, and AC conducted laboratory analysis. DH and KG conducted bioinformatics. AC and DH ran statistical analysis. LZ, EC, DH, PM, and AC wrote the manuscript. All authors contributed to the article and approved the submitted version.

## Conflict of Interest

The authors declare that the research was conducted in the absence of any commercial or financial relationships that could be construed as a potential conflict of interest.

## Supplementary Material

Supplementary Figure 1 Non-metric multidimensional scaling (NMDS) of the Bray–Curtis dissimilarities for the archaeal and bacterial solid-associated rumen samples by substrate and treatment.
