# Supplementary figures and images for: Ozone Decreased Enteric Methane Production by 20% in an in vitro Rumen Fermentation System
Source: Front Microbiol. 2020 Nov 2;11:571537. doi: 10.3389/fmicb.2020.571537 (PMC7667233; doi:10.3389/fmicb.2020.571537)

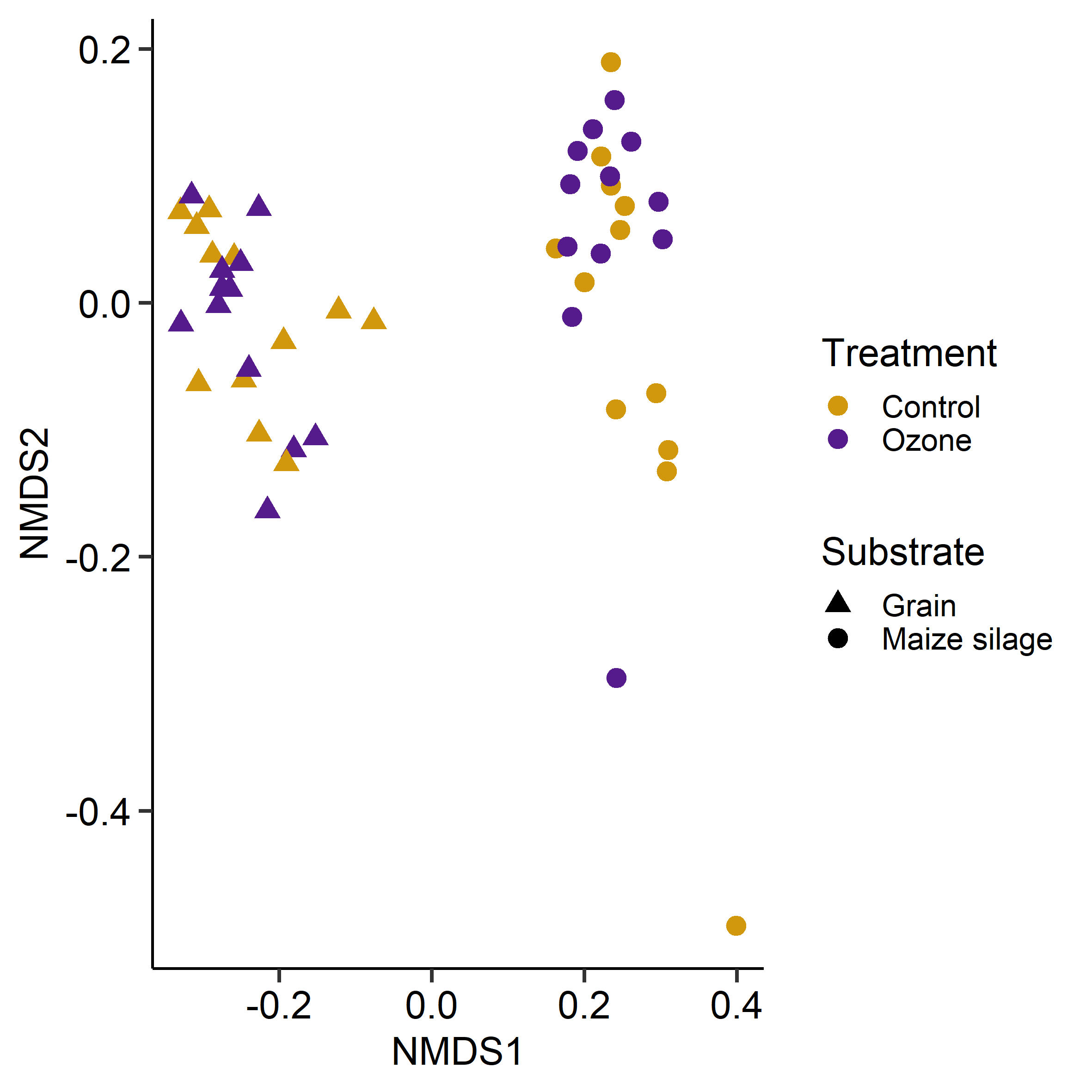

Supplement: Supplementary Figure 1 — Non-metric multidimensional scaling (NMDS) of the Bray–Curtis dissimilarities for the archaeal and bacterial solid-associated rumen samples by substrate and treatment. [file Image_1.TIFF]
