# Supplementary material for: Ozone Decreased Enteric Methane Production by 20% in an in vitro Rumen Fermentation System
Source: Front Microbiol. 2020 Nov 2;11:571537. doi: 10.3389/fmicb.2020.571537 (PMC7667233; doi:10.3389/fmicb.2020.571537)
Supplement: Supplementary file 2 [file Table_1.DOCX]

**Supplementary Table S1.** Effect of ozone on richness (number of OTUs) and diversity measures on liquid-associated microbes (LAM) for each sampling day.

| **LAM** | Day | Control | Ozone | SEM | *P*-value |
| --- | --- | --- | --- | --- | --- |
| Number of OTUs | 5 | 749 | 630 | 68.4 | 0.25 |
| Shannon diversity index | 5 | 4.9 | 4.7 | 0.16 | 0.30 |
| Inverse Simpsons diversity | 5 | 31.6 | 25.4 | 5.00 | 0.40 |
|  |  |  |  |  |  |
| Number of OTUs | 10 | 478 | 459 | 62.5 | 0.84 |
| Shannon diversity index | 10 | 4.2 | 4.2 | 0.24 | 0.95 |
| Inverse Simpsons diversity | 10 | 20.8 | 21.9 | 57.41 | 0.96 |

SEM, standard error of the means.
